# The New Orleans Healthy Default Beverage Policy and Beverage Ordering Among Children

**DOI:** 10.1001/jamanetworkopen.2026.17749

**Published:** 2026-06-10

**Authors:** Melissa Fuster, Yin Wang, Charles Stoecker, María M. Muñoz, Donald Rose, Lisa P. Hofmann, Megan Knapp

**Affiliations:** 1 Social, Behavioral, and Population Sciences Department, Tulane University Celia Scott Weatherhead School of Public Health and Tropical Medicine, New Orleans, Louisiana; 2 Department of Social Science and Health Management, College of Public Health, Zhengzhou University, Zhengzhou, Henan, China; 3 Department of Health Policy and Management, Tulane University Celia Scott Weatherhead School of Public Health and Tropical Medicine, New Orleans, Louisiana; 4 Department of Public Health Sciences, Xavier University of Louisiana, New Orleans

## Abstract

**Importance:**

Sugar-sweetened beverages (SSBs) are the leading source of added sugar in US children’s diets and are linked to increased obesity risk, and restaurants are critical settings for interventions to reduce SSB consumption. Local menu ordinances requiring default healthier beverages (HDB) are gaining in popularity, but to date, there has been no rigorous evaluation of their effectiveness.

**Objective:**

To evaluate the outcomes of an HDB ordinance on beverage ordering for children in restaurants.

**Design, Setting, and Participants:**

Difference-in-differences study using caregiver-reported survey data before and after policy implementation (September 2022 and 2024, respectively) in 2 urban parishes in Louisiana: New Orleans (intervention city) and Baton Rouge (comparison city). Parents and caregivers (≥18 years) of children aged 2 to 12 years who had ordered or eaten at a restaurant with their child in the past 30 days were surveyed.

**Exposure:**

Implementation of the New Orleans Healthy Kids’ Meal Beverage Ordinance, which mandates that only water, unflavored or low-fat milk, or 100% juice be offered as default beverages with children’s meals.

**Main Outcomes and Measures:**

The primary outcome was ordering of a healthy default beverage with the most recent children’s restaurant meal. Secondary outcomes included beverage volume, sugar and calorie intake, and selection of beverage types. Models were weighted and adjusted for demographics, dining mode, and caregiver attitudes.

**Results:**

A total of 1006 caregivers completed the baseline survey (736 [73.2%] female; 506 in New Orleans, 500 in Baton Rouge), and 1131 completed the postpolicy survey (548 [48.5%] female; 556 in New Orleans, 575 in Baton Rouge). Healthy beverage ordering decreased in both cities, but comparison of before-and-after differences between the 2 cities indicates the ordinance was associated with higher odds of ordering a healthy beverage (adjusted odds ratio [aOR], 2.10; 95% CI, 1.16 to 3.83; *P* = .02), attenuating the ordering decline in New Orleans. Sugar intake decreased by 5.6 g (95% CI, −9.6 to −1.5 g; *P* = .007), and calorie intake declined by 34.0 kcal (95% CI, −59.1 to −8.9 kcal; *P* = .008). The odds of ordering water increased (aOR, 2.08; 95% CI, 1.12 to 3.85), while the odds of ordering 100% juice decreased (aOR, 0.59; 95% CI, 0.36 to 0.96). No significant changes were observed for SSBs or beverage volume.

**Conclusions and Relevance:**

In this survey study, the New Orleans HDB ordinance was associated with modest but meaningful improvements in children’s beverage ordering, supporting default-based policies as a tool for promoting healthier dietary behaviors.

## Key Points

**Question:**

Was the 2023 New Orleans restaurant healthy default beverage (HDB) ordinance associated with changes in children’s beverage orders?

**Findings:**

In this survey study of 2137 parents and caregivers in New Orleans (policy city) and Baton Rouge (comparison city), implementation of the HDB ordinance was associated with higher odds of ordering a healthy beverage and reduced sugar and calorie intake among children.

**Meaning:**

These findings suggest HDB policies may support healthier dietary behaviors in children and represent a feasible strategy for obesity prevention in restaurant settings.

## Teaser

This survey study evalutes the association of a healthy default beverage ordinance with beverage ordering for children in restaurants.

## Introduction

Sugar-sweetened beverages (SSBs), including sodas, as well as fruit-flavored, sports, and energy drinks, sweetened waters, tea, and coffee, are the highest source of added sugar in children’s diets aged 2 to 12 years.^1^ Reducing added sugar consumption can lower the prevalence of childhood obesity, which disproportionately affects non-Hispanic Black and low-income children.^2,3^ Restaurants are a key environment for healthier food and beverage policies. It is estimated that US consumers spend almost half of their food and beverage budget outside the home, and almost one-third of calories from food and beverages are consumed outside the home.^4^ Restaurant consumption among children has been linked to higher intakes of sugar, fat, saturated fat, and sodium, with sugar-sweetened beverages contributing a notable share of excess calories.^5,6^ Children’s meals (ie, menu options tailored for children where a whole or combination meal including main dish, side and/or dessert is priced together), that include SSBs have, on average, 179 calories more than meals without SSBs.^7^

Childhood obesity is of particular concern in Louisiana. As of 2022, this Southern state had the third-highest childhood obesity rate at 22.2% (among those aged 10-17 years) and fourth-highest among adults at 38.1%.^8^ In New Orleans, the largest city in the state, a healthy default beverage (HDB) policy took effect in January 2023, requiring restaurants to offer water, unflavored nonfat or 1% milk, or 100% juice (up to 6.75 oz) as the default drink with children’s meals. This policy is especially significant in a city where sugary drink consumption is high and nutrition-related health disparities are deeply entrenched.^6,9,10^ By targeting restaurant defaults, the ordinance represents a structural, upstream intervention as it changes the choice architecture within restaurants with the potential to shift dietary norms and reduce chronic disease risk at the population level.^11^

HDB policies are among the most commonly proposed and enacted beverage policies in the US to curb SSB intake for children.^12^ These policies build on the fact that consumers tend to choose the standard menu offerings.^13^ The policy does not prohibit parents from ordering an SSB for their child, but they may make the healthier choice an easier and, hence, likelier choice.^14,15^ Yet, evaluation efforts that address the effectiveness of changing choices are lacking. Studies explicitly addressing real-world HDB policies have focused on implementation and compliance, mostly in fast-food settings.^16,17,18,19^ Addressing this significant research gap, this study examines changes in the treatment and a comparison city before and after the policy implementation. Specifically, the objective of the present study was to examine changes in restaurant beverage consumption among children aged 2 to 12 years in New Orleans, Louisiana and Baton Rouge, Louisiana (comparison city) after the implementation of the HDB policy.

## Methods

For this survey study, we employed a difference-in-differences (DiD) design to evaluate the association between the New Orleans Healthy Kids’ Meal Beverage Ordinance and children’s beverage ordering behaviors together with a meal. Data were collected using an online survey distributed in September 2022 (at baseline) and September 2024 (after policy implementation) to caregivers of children aged 2 to 12 years in New Orleans or Baton Rouge. The analysis compared outcomes in New Orleans (the treated city) with those in Baton Rouge (the comparison city) before and after policy implementation. The study was classified as exempt from the need for review and written informed consent by the Xavier University institutional review board because the study posed no more than minimal risk and met the criteria for exemption category 2 as a study involving survey procedures with no risk of a break in anonymity. Our reporting follows the Strengthening the Reporting of Observational Studies in Epidemiology (STROBE) reporting guideline.

### Study Sample

This study used a repeated cross-sectional approach. The survey was distributed by an online marketing research firm (Centiment), which uses an opt-in online panel of respondents recruited via social media and affiliated networks based on study-specific demographic and behavioral characteristics (the survey is reproduced in the eAppendix in Supplement 1). In this study, inclusion criteria for participants included being 18 years or older, living in 1 of the target cities (New Orleans or Baton Rouge), being a parent or caregiver to a child between the ages of 2 and 12 years, and having ordered from or eaten at a restaurant with their child in 1 of the target cities within the last 30 days. We used this age range because many restaurants limit children’s menus to those up to 12 years of age.^20^ Participants who completed the survey were compensated directly by Centiment. A total of 1006 caregivers completed the baseline survey (506 from New Orleans and 500 from Baton Rouge), and 1131 caregivers completed the postpolicy survey (556 from New Orleans and 575 from Baton Rouge), who shared data on a total of 4904 meals with children (dine-in and take-out) across the 2 cities and waves. Our preliminary power calculations indicated 500 children from each city would be enough to capture differences as small as 23.8 calories (α = .05, power, 80%, mean [SD], 128.7 [134.2]).^7^

### Data Collection

Data were collected using an online survey developed by the investigators using Qualtrics and informed by previous research.^6,21,22^ We designed the survey to be completed within 10 to 15 minutes. The survey collected detailed information about (1) the most recent restaurant meal eaten in a restaurant and (2) for a recent meal eaten out of the restaurant (take-out, delivery, or drive-through). If more than 1 child aged 2 to 12 years was present at the eating occasion, the respondent provided information for 2 children: the oldest and the youngest. For each meal, we collected the type of meal ordered (eg, meal from the children’s menu, regular menu, or shared family meal) and information about the drink ordered, including type of drink (eg, water, SSB, and so forth), portion size ordered, amount consumed, and whether the drink included ice. To facilitate accurate estimation of consumption amounts, participants were provided with standardized visual aids depicting commonly available beverages from restaurants that reflect typical portion sizes and formats encountered in real-world contexts.

Additional questions included respondent and child demographics and attitudes and practices related to SSBs and restaurant meals. Demographic questions were assessed using standard questions.^21,22^ We examined respondent race and ethnicity using a multiple selection question with alphabetized categories and an open-response option. We assessed attitudes toward SSBs using a 5-point Likert question assessing the level of agreement with the following phrase: “Sugary-sweetened drinks are an important aspect of family meals.” This item was developed based on previous studies^23,24^ and shown to have a positive association with higher SSB intake frequency among children.^6^ Eating out frequency was measured for the past 30 days, including meals consumed at a restaurant or purchased at a restaurant as take-out or delivery, with frequency choices ranging from once a month to more than once a day. The survey used digital fingerprinting technology to ensure that each survey was submitted by a unique participant and applied measures (attention checks and response validation) to ensure respondents were fully engaged.

The survey was first pretested among a small group of 10 caregivers, to assess how the questions were answered and interpreted to ensure clarity. Second, the revised survey underwent a soft launch with a pilot sample of 20 respondents before full implementation. Participants received an invitation that included minimal study information to avoid selection bias. The survey landing page included a consent information sheet confirmed by proceeding to the survey. Documentation of written informed consent was waived.

### Outcome Measures

The primary outcome was a binary indicator of whether an HDB was ordered based on the most recent restaurant meal. This was derived from the beverage type reported. HDB compliant beverages were defined according to the policy as: (1) unflavored, unsweetened, noncarbonated water; (2) plain, pasteurized nonfat or 1% cow’s milk with no added sweeteners or flavorings or nondairy milk alternatives containing no more than 130 calories per serving; or (3) 100% juice or juice diluted with water or carbonated water, with no added sweeteners and a serving size not exceeding 6.75 ounces.^25^ We also examined the ordinance’s association with several secondary outcomes: volume, total sugar, and calorie intake associated with the beverage volume consumed, and the types of beverages selected—specifically, SSBs and HDB-compliant beverages. Caloric and nutrient data were derived from the US Department of Agricuture’s Food and Nutrient Database for Dietary Studies (FNDDS) nutrient values (2019-2020). These measures were designed to capture behavioral changes associated with the policy. Covariates for our analysis included demographics (child age and sex, self-reported caregiver race and ethnicity, sex, educational attainment, and household income), type of eating occasion (eat-in vs take-out), frequency of eating out, and SSB-related attitude selected based on past research and preliminary analyses.^6,21,22^

### Statistical Analysis

We used logit regression models to estimate the associations between the ordinance and binary outcomes and linear regression models for continuous outcomes. In both cases, models included indicator variables for the main effects of city (intervention vs comparison) and time period (after vs before), as well as an interaction term between the 2, which is the policy effect. All models were weighted using survey weights to ensure representativeness of the sampled population. Centiment conducts surveys based on designated market areas. We obtained race and ethnicity compositions for each market area from the 5-year American Community Survey using 2022 for the prepolicy wave and 2024 for the postpolicy wave. We then used Stata’s svycal regress routine to generate survey weights that matched the race and ethnicity categories weights for each city and survey year. Because the outcomes analyzed in this study are measured at the child dining case level, caregiver weights were apportioned to the child observations. Specifically, if a caregiver reported 2 child dining cases, each child was assigned half of that caregiver’s weight; if only 1 child dining case was reported, the child was assigned the full caregiver weight. Models controlled for sociodemographic measures, including child sex and age, caregiver sex, race, educational attainment, household income, and the additional covariates described previously. Cluster-robust standard errors were estimated at the zip code level to account for serial correlation in dining behaviors as well as intrahousehold correlations between children. All analysis was conducted with Stata version 16 (StataCorp). Robust SEs were obtained by using the cluster option in the corresponding regression commands.

## Results

A total of 1006 caregivers completed the baseline survey (736 [73.2%] female; 506 in New Orleans, 500 in Baton Rouge), and 1131 completed the postpolicy survey (548 [48.5%] female; 556 in New Orleans, 575 in Baton Rouge). We surveyed 1921 caregivers across the 2 waves, of whom 1876 reported dine-in occasions and 1839 reported take-out occasions for their children. Among dine-in occasions, 1212 caregivers reported having only 1 child, while 664 reported having 2 or more children. For take-out occasions, 1314 caregivers reported having only 1 child and 525 reported on 2 children. In total, 2540 meals with children were included in the dine-in sample and 2364 in the take-out sample. Table 1 presents weighted descriptive statistics for the analytic sample, stratified by parish and survey wave. Across both periods, the distributions of child and caregiver sociodemographic characteristics, as well as dining-related behaviors, were largely comparable between New Orleans and Baton Rouge, although the baseline samples in both cities included more female caregivers with lower educational attainment and those less likely to agree that SSBs are important in family meals than the postsample. The proportion of healthy default beverages ordered was lower in the postpolicy survey wave compared with the prepolicy wave in both parishes, with a larger difference observed in Baton Rouge (from 326 participants [23.9%] to 111 [9.8]%) compared with New Orleans (from 310 participants [23.0%] to 181 [17.0]).

**Table 1.** Characteristics of Caregivers and Children, Prepolicy and Postpolicy Implementation (January 2023)

Characteristic	Participants, No. (%)^a^			
	Preperiod (September 2022)		Postperiod (September 2024)	
	New Orleans (n = 1350)	Baton Rouge (n = 1363)	New Orleans (n = 1064)	Baton Rouge (n = 1127)
Child sex				
Female	669 (49.53)	676 (49.59)	415 (38.99)	523 (46.44)
Male	681 (50.47)	687 (50.41)	649 (61.01)	604 (53.56)
Child age, y				
<5	370 (27.41)	440 (32.28)	250 (23.51)	325 (28.81)
5-9	559 (41.42)	523 (38.34)	394 (37.07)	546 (48.44)
>9	421 (31.17)	400 (29.38)	419 (39.42)	256 (22.75)
Caregiver sex				
Nonfemale	360 (26.70)	322 (23.63)	581 (54.64)	477 (42.33)
Female	990 (73.30)	1041 (76.37)	483 (45.36)	650 (57.67)
Caregiver race				
Black	396 (29.32)	466 (34.17)	338 (31.77)	391 (34.70)
White	736 (54.50)	739 (54.22)	567 (53.32)	609 (54.07)
Other^b^	218 (16.18)	158 (11.62)	159 (14.91)	127 (11.23)
Caregiver’s educational attainment				
High school or less	377 (27.92)	456 (33.46)	198 (18.65)	285 (25.26)
Some college	397 (29.38)	469 (34.40)	274 (25.74)	310 (27.54)
Bachelor’s degree or higher	576 (42.70)	438 (32.14)	592 (55.60)	532 (47.20)
Household income level, US $				
<100 000	1053 (77.98)	1085 (79.57)	790 (74.23)	785 (69.67)
≥100 000	253 (18.72)	224 (16.42)	258 (24.27)	317 (28.09)
Prefer not to say	45 (3.30)	55 (4.01)	16 (1.51)	25 (2.25)
Caregiver’s attitude toward SSBs (SSBs important in family meals)				
Disagree	621 (46.03)	607 (44.51)	285 (26.76)	343 (30.43)
Neutral	406 (30.07)	503 (36.87)	225 (21.19)	319 (28.29)
Agree	323 (23.89)	254 (18.62)	554 (52.06)	465 (41.28)
Restaurant visit frequency				
Monthly	564 (41.78)	584 (42.84)	425 (39.95)	471 (41.78)
Weekly	337 (24.93)	373 (27.40)	217 (20.38)	266 (23.64)
≥2/wk	449 (33.29)	406 (29.77)	422 (32.67)	390 (34.58)
Dining mode				
Take-out	673 (49.82)	673 (49.41)	526 (49.43)	552 (49.02)
Dine-in	677 (50.18)	690 (50.59)	538 (50.57)	575 (50.98)
Beverage selection				
Nondefault beverages	1040 (77.03)	1037 (76.08)	883 (82.98)	1016 (90.17)
Healthy default beverages	310 (22.97)	326 (23.92)	181 (17.02)	111 (9.83)
SSB	911 (67.52)	895 (65.65)	703 (66.08)	754 (66.87)
Water	187 (13.82)	191 (14.01)	180 (16.88)	103 (9.14)
Milk	14 (1.06)	32 (2.38)	10 (0.98)	18 (1.59)
Juice	191 (14.14)	192 (14.06)	119 (11.20)	205 (18.22)
Beverage volume ordered, mean (SD), oz	13.56 (7.05)	13.25 (7.10)	16.67 (6.04)	15.53 (5.43)
SSB volume ordered, mean (SD), oz	9.90 (8.97)	9.46 (8.92)	11.25 (9.54)	10.68 (8.87)
Sugar consumed, mean (SD), g	27.66 (27.30)	24.54 (22.81)	30.79 (29.22)	31.66 (24.07)
Energy consumed, mean (SD), kcal	133.64 (148.02)	121.57 (131.30)	143.47 (145.51)	159.51 (156.18)

Abbreviation: SSB, sugar-sweetened beverage.

^a^ Weighted analysis. Percentages may not total 100 because of rounding.

^b^ Other race included Asian, Hispanic or Latino, American Indian, Native Hawaiian or Pacific Islander, and multiracial individuals.

We employed a series of DiD logit regression models to estimate the association between the New Orleans Healthy Kids’ Meal Beverage Ordinance and the likelihood of ordering a healthy default beverage (Table 2). While the unadjusted model does not yield a statistically significant estimate, the odds ratio (OR) for the policy outcome (DiD coefficient = 1.98) was numerically similar to those in the adjusted models. In the DiD framework, these ORs indicate that the change in the odds of ordering an HDB in New Orleans between the prepolicy and postpolicy periods was approximately twice as large as the corresponding change observed in Baton Rouge. Comparison of before-and-after differences between the 2 cities indicates the ordinance was associated with higher odds of ordering a healthy beverage (adjusted odds ratio [aOR], 2.10; 95% CI, 1.16 to 3.83; *P* = .02), attenuating the ordering decline in New Orleans. We also estimated the ordinance outcomes on the probability scale (the marginal effect of the DiD interaction term between the postperiod indicator and the treatment area indicator), which measures the difference between the estimated postpolicy probability of ordering an HDB in New Orleans and the counterfactual probability if without the policy. Given the declining trend in the HDB ordering in both parishes (Table 1), the results suggest that the ordinance attenuated the decline in HDB selection in New Orleans by approximately 11 percentage points relative to Baton Rouge.

**Table 2.** Outcomes of the Ordinance for Healthy Beverage Ordering^a^

Statistic	Unadjusted (n = 4904)	Adjusted model 1 (n = 4904)	Adjusted model 2 (n = 4904)	Adjusted model 3 (n = 4904)
Post × treat (OR-scale policy outcome)^b^	1.98 (0.86-4.59)	1.99 (1.09-3.63)	2.01 (1.12-3.62)	2.10 (1.16-3.83)
*P* value	.11	.02	.02	.02
Post^c^	0.35 (0.25-0.47)	0.30 (0.22-0.42)	0.30 (0.21-0.42)	0.29 (0.21-0.41)
*P* value	<.001	<.001	<.001	<.001
Treat^d^	0.95 (0.70-1.29)	0.93 (0.69-1.24)	0.92 (0.68-1.23)	0.92 (0.68-1.24)
*P* value	.73	.61	.55	.59
Probability-scale policy outcome^e^	0.11	0.11	0.11	0.11
Baseline outcome^f^	0.23	0.23	0.23	0.23
Outcome, %^g^	49	47	48	50
Sociodemographics^h^	No	Yes	Yes	Yes
SSB attitude^i^	No	No	Yes	Yes
Restaurant use type^j^	No	No	No	Yes

Abbreviations: OR, odds ratio; SSB, sugar-sweetened beverage.

^a^ Each column presents results from a logit regression model controlled for different sets of covariates, as noted in the corresponding variable group row, using a binary indicator of ordering a healthy beverage as the dependent variable.

^b^ The difference-in-differences interaction term between postpolicy indicator and treatment area indicator, the coefficient of which indicates the policy outcome on the odds ratio scale.

^c^ The postpolicy period indicator.

^d^ The treatment area indicator (New Orleans).

^e^ The policy outcome on the probability scale, estimated as the marginal effect of the difference-in-differences interaction term, which translates the OR-scale policy outcome into percentage-point changes in probability.

^f^ Mean value of the outcome variable in the treatment area (New Orleans) in the prepolicy period.

^g^ The probability-scale policy outcome as a proportion of the baseline outcome.

^h^ Including child sex, child age, caregiver sex, caregiver race, caregiver educational attainment, and household income level.

^i^ Caregivers’ attitude toward the statement “Sugar-sweetened beverages are an important aspect of family meals.”

^j^ Including restaurant visit frequency and dining mode (dine-in or take-out).

We found no statistically significant association between the ordinance and the total volume of beverages ordered or the volume of SSBs ordered (Table 3). However, the ordinance was associated with statistically significant reductions in sugar and calorie intake from beverages. Specifically, total sugar intake declined by 5.6 g (95% CI, –9.6 to –1.5; *P* = .007), and energy intake decreased by 34.0 kilocalories (95% CI, –59.1 to –8.9; *P* = .008), corresponding to reductions of approximately 20% and 25%, respectively, relative to baseline levels.

**Table 3.** Outcomes of the Ordinance for Beverage Volume, Sugar, and Calorie Intake^a^

Statistic	Volume of any beverage, oz (n = 4904)	Volume of SSBs, oz (n = 4904)	Sugar intake, g (n = 4904)	Energy intake, kilocalories (n = 4904)
Post × treat (policy outcome in outcome unit)^b^	0.11 (−1.13 to 1.34)	−0.45 (−1.81 to 0.91)	−5.55 (−9.57 to −1.53)	−33.99 (−59.09 to −8.89)
*P* value	.86	.51	.007	.008
Post^c^	2.25 (1.47 to 3.03)	1.54 (0.47 to 2.62)	8.25 (5.09 to 11.41)	42.54 (19.71 to 65.38)
*P* value	<.001	.005	<.001	<.001
Treat^d^	0.12 (−0.51 to 0.75)	0.38 (−0.45 to 1.22)	3.11 (0.89 to 5.33)	11.76 (−1.18 to 24.70)
*P* value	.71	.37	.006	.07
Baseline outcome^e^	13.56	9.90	27.66	133.64
Outcome, %^f^	1	−5	−20	−25

Abbreviation: SSB, sugar-sweetened beverage.

^a^ Each column presents results from a linear regression model controlled for covariates for demographics (child sex, child age, caregiver sex, caregiver race, caregiver educational attainment, and household income level), caregivers’ SSB attitude (level of agreement with the statement “Sugar-sweetened beverages are an important aspect of family meals.”), and restaurant use variables (use frequency and dining mode: dine-in vs take-out). The outcome is indicated on the column title.

^b^ The difference-in-differences interaction term between postpolicy indicator and treatment area indicator, the coefficient of which indicates the policy outcome in the unit of the outcome.

^c^ The postpolicy period indicator.

^d^ The treatment area indicator (New Orleans).

^e^ Mean value of the outcome variable in the treatment area (New Orleans) in the prepolicy period.

^f^ The policy outcome as a proportion of the baseline outcome.

The ordinance was not significantly associated with changes in SSB or HDB-compliant milk ordering (Table 4). In contrast, statistically significant effects were found for water and 100% juice. The odds of selecting water were higher (OR, 2.08; 95% CI, 1.12 to 3.85; *P* = .02), while the odds of selecting 100% juice were lower (OR, 0.59; 95% CI, 0.36 to 0.96; *P* = .04). These effects correspond to a 66% increase in water selection and a 39% decrease in juice selection relative to baseline probabilities, respectively.

**Table 4.** Outcomes of the Ordinance on Beverage Type Selection^a^

Statistic	SSB (n = 4904)	Water (n = 4904)	Milk (n = 4772)	Juice (n = 4904)
Post × treat (OR-scale policy outcome)^b^	0.85 (0.57 to 1.26)	2.08 (1.12 to 3.85)	1.51 (0.40 to 5.65)	0.59 (0.36 to 0.96)
*P* value	.41	.02	.54	.04
Post^c^	1.16 (0.87 to 1.54)	0.52 (0.34 to 0.80)	0.54 (0.19 to 1.53)	1.49 (1.06 to 2.10)
*P* value	.31	.003	.25	.02
Treat^d^	1.1 (0.85 to 1.43)	0.92 (0.61 to 1.38)	0.41 (0.18 to 0.91)	1.12 (0.83 to 1.50)
*P* value	.46	.69	.03	.46
Probability-scale policy outcome^e^	−0.04	0.09	0.01	−0.06
Baseline outcome^f^	0.68	0.14	0.01	0.14
Outcome, %^g^	−5	66	59	−39

Abbreviations: OR, odds ratio; SSB, sugar-sweetened beverage.

^a^ Each column presents results from a logit regression model controlled for covariates for demographics (child sex, child age, caregiver sex, caregiver race, caregiver educational attainment, and household income level), caregivers’ SSB attitude (level of agreement with the statement “Sugar-sweetened beverages are an important aspect of family meals.”), and restaurant use variables (use frequency and dining mode: dine-in vs take-out). The outcome is a binary indicator of whether a corresponding type of beverage was selected.

^b^ The difference-in-differences interaction term between postpolicy indicator and treatment area indicator, the coefficient of which indicates the policy outcome on the odds ratio scale.

^c^ The postpolicy period indicator.

^d^ The treatment area indicator (New Orleans).

^e^ The policy outcome on the probability scale, estimated as the marginal effect of the difference-in-differences interaction term, which translates the OR-scale policy outcome into percentage-point changes in probability.

^f^ Mean value of the outcome variable in the treatment area (New Orleans) in the prepolicy period.

^g^ The probability-scale policy outcome as a proportion of the baseline outcome.

## Discussion

To our knowledge, this study is the first to use a controlled DiD design to examine actual changes in beverage purchases and intake resulting from an HDB ordinance. We show that the policy was associated with a significantly higher likelihood of ordering a healthy beverage, as defined by the policy, in restaurant settings. Previous evaluations have focused on compliance, showcasing small but positive impacts on restaurant offerings and menu design.^13,19,26,27^ A limited number of studies have documented changes in orders in response to menus featuring HDB in response to voluntary changes (not policy mandates), documenting increases in orders of healthier drinks (HDB-compliant milk and 100% juice) and decreases in energy intake from the beverages.^28,29^

Our study also showed that, although the absolute proportion of HDB choices declined in both cities during the study period, the decrease was notably smaller in New Orleans. The overall decline in HDB choices across waves may be due to industry response to the increased popularity of HDB choices, as the restaurant industry continues to market sugary beverages to children (eg, branding of SSB cups with movie characters).^30,31,32^ While our results suggest that the New Orleans HDB policy may have been protective against potential external factors, these results underscore the need for additional regulation and efforts, as no single policy can serve as a silver bullet against complex issues, such as childhood obesity and the associated SSB intake.

We also found a significant shift in the types of healthy beverages selected. Notably, the odds of water selection increased. This suggests that the policy may have helped reframe water as a more socially normative or accessible option. However, while the study did not find significant differences in SSB selection, we found a decrease in 100% juice selection. These shifts may help explain the observed reductions in sugar and caloric intake from beverages among children in New Orleans relative to Baton Rouge. While 100% juice is considered healthier than SSBs, it is still high in sugar. These results also indicate that the policy is addressing the problem it intends to address, sugar consumption, with these reductions potentially contributing to improved health outcomes in the long-term, should the trend continue.

Despite these promising results, it is important to recognize that HDB policies alone are not sufficient to shift population-level consumption patterns. The relatively modest changes observed—particularly in the absence of stronger reductions in SSB ordering—highlight the limitations of this approach, especially in food environments where structural, cultural, and commercial influences continue to normalize sugary beverages. Given that SSBs remain the largest source of added sugar in children’s diets and disproportionately affect minoritized and low-income youth, multifaceted strategies will likely be needed to address these persistent inequities. Complementary interventions, including regulation of marketing to children, fiscal policies (eg, SSB taxes), and the normalization of healthier beverages could enhance the effectiveness of default policies in high-burden communities.

Our data capture both on-site and off-site consumption, including take-out and delivery orders, increasingly common modes of food acquisition that are often overlooked in restaurant-based research. By directly capturing caregiver-reported beverage selection and consumption behaviors using a pre-post design with a comparable city, this study adds to a limited but growing evidence base on the behavioral outcomes of restaurant-based nutrition policies, going beyond availability and marketing to assess how defaults may shape actual choices. This study also contributes important insights into policy implementation in Southern cities with high rates of childhood obesity. Louisiana ranks among the highest in childhood and adult obesity rates, and New Orleans presents a critical context for evaluating interventions that may advance health equity. That the HDB policy had a measurable outcome in this setting underscores its relevance for other jurisdictions with similar demographic and health profiles.

### Limitations

This study has limitations. We used a nonprobability online panel sample. Therefore, our findings may be not representative of the population. We sought to address this through the use of sample weights. Moreover, as a robustness check on these weights, we reran our main analyses in Table 2 without applying the weights and the results were substantively consistent (see eTable in Supplement 1). While our sampling approach limits generalizability, compared with intercept-style convenience samples, online panel-based recruitment allows for broader geographic reach and targeted eligibility criteria. In addition, the DiD design may be less sensitive to selection bias than cross-sectional estimates as both treatment and control groups were subject to the same selection effects. We also relied on self-reported data, which may be subject to social desirability and recall bias. However, we see no reason why this would operate more strongly in New Orleans than in Baton Rouge. While social desirability may be reduced in online surveys, we sought to minimize recall bias by asking about the last meal consumed and limiting our sample to those who had purchased a meal within the last 30 days. Further, this study is based on repeated cross-sections rather than longitudinal data collection. Thus, different parents are represented in each survey wave. We addressed this problem using statistical adjustment with a robust set of demographic, socioeconomic, attitudinal, and behavioral variables, although we realize that a longitudinal design, if possible, would have been stronger. We also recognize the need for future research to examine the longer-term effects of this policy, as the postassessment took place 20 months after policy implementation.

## Conclusions

In this survey study of the New Orleans Healthy Kids’ Meal Beverage Ordinance, we showed that the policy was associated with a higher likelihood of healthy beverage selection and lower sugar and calorie consumption among children ordering meals in restaurants after the HDB policy implementation. While the magnitude of change was modest, these results provide evidence that default-based policies may influence consumer behavior in ways that support public health goals. Given the persistent disparities in diet-related disease and the role of restaurant environments in shaping children’s dietary patterns, HDB policies may serve as a useful component of broader strategies to reduce added sugar intake and advance health equity. Future work should address the long-term impact of the policy in health outcomes, as well as how to expand and support these efforts, for instance, addressing the nutritional value of children’s meals as well as expanding these efforts beyond this locality, to the state, and integrating HDB ordinances into comprehensive food and nutrition policy frameworks.

## Notes

Supplement 1. eTable. Impact of the Ordinance on Healthy Beverage Ordering eAppendix. New Orleans Healthy Default Beverage Ordinance Evaluation: Survey Instrument

Supplement 2. Data Sharing Statement
