# Supplementary material for: The New Orleans Healthy Default Beverage Policy and Beverage Ordering Among Children
Source: JAMA Netw Open. 2026 Jun 10;9(6):e2617749. doi: 10.1001/jamanetworkopen.2026.17749 (PMC13254729; doi:10.1001/jamanetworkopen.2026.17749)
Supplement: Supplement 1. — eTable. Impact of the Ordinance on Healthy Beverage Ordering eAppendix. New Orleans Healthy Default Beverage Ordinance Evaluation: Survey Instrument [file jamanetwopen-e2617749-s001.pdf]

Supplemental Online Content

Fuster M, Wang Y, Stoecker C, et al. The New Orleans healthy default beverage policy and beverage ordering among children. *JAMA Netw Open*. 2026;9(6):e2617749. doi:10.1001/jamanetworkopen.2026.17749

eTable. Impact of the Ordinance on Healthy Beverage Ordering
eAppendix. New Orleans Healthy Default Beverage Ordinance Evaluation: Survey Instrument

This supplemental material has been provided by the authors to give readers additional information about their work.

© 2026 Fuster M et al. *JAMA Network Open*.

eTable. Impact of the Ordinance on Healthy Beverage Ordering (No weights Applied in Analysis)

	Unadjusted	Adjusted Model 1	Adjusted Model 2	Adjusted Model 3
Post×Treat (OR-Scale Policy Impact)	1.87	1.95*	1.96*	1.97*
	[0.87, 4.04]	[1.05, 3.61]	[1.07, 3.59]	[1.09, 3.54]
	(0.11)	(0.03)	(0.03)	(0.02)
Post	0.41**	0.37**	0.37**	0.37**
	[0.30, 0.56]	[0.27, 0.51]	[0.27, 0.52]	[0.27, 0.52]
	(<0.001)	(<0.001)	(<0.001)	(<0.001)
Treat	1.05	1.03	1.02	1.03
	[0.83, 1.32]	[0.82, 1.29]	[0.81, 1.28]	[0.82, 1.30]
	(0.69)	(0.79)	(0.85)	(0.80)
Probability-Scale Policy Impact	0.10	0.11	0.11	0.11
Baseline Outcome	0.23	0.23	0.23	0.23
Impact (%)	45	46	47	46
Sociodemographics	No	Yes	Yes	Yes
SSB Attitude	No	No	Yes	Yes
Restaurant Use Type	No	No	No	Yes
N	4904	4904	4904	4904

Notes: 95% CIs are reported in square brackets, and p-values are reported in parentheses. Parameter definitions are identical to those used in the updated Table 2 in the manuscript.
* $p<.05$, ** $p<.01$.

© 2026 Fuster M et al. *JAMA Network Open*.

eAppendix. New Orleans Healthy Default Beverage Ordinance Evaluation: Survey Instrument

This instrument reflects the survey as administered via an online platform and has been formatted for readability. Minor differences in formatting, skip logic, and question display may exist between this document and the programmed survey. The survey was administered using programmed skip logic and adaptive display; therefore, the order and visibility of items may vary from this static version. This instrument is provided for transparency and research purposes only and should not be reproduced or used without permission. The original instrument was a separate survey for the intervention city (New Orleans) and the comparison city (Baton Rouge). For the purposes of this document, these have been merged. Additional notes are added for clarification using red font. Questions that were added in the follow-up survey are denoted with an asterisk.

Q1 Thank you for your help with this important survey! We are seeking to learn more about children's beverage consumption when eating out. The study has been approved by the Institutional Review Board at Xavier University of Louisiana (Protocol #894). The survey may take between 10-15 minutes. Your participation is voluntary. You may choose to not answer any questions. Your answers will be kept anonymous. If you have any questions or concerns, please reach out to the project directors, Dr. Megan Knapp (mknapp@xula.edu) and Dr. Melissa Fuster (mfuster@tulane.edu). If you agree to participate, please click on the arrow below.

Q2 Are you 18 years of age or older?

○ Yes

○ No

Skip To: End of Survey If Are you 18 years of age or older? = No

Q3 Do you live in or near Baton Rouge or New Orleans (dependent upon survey location)?

○ Yes

○ No

Skip To: End of Survey If Do you live in or near Baton Rouge or New Orleans? = No

Q4 What is your Zip Code? ____

Skip To: End of Survey If Condition: What is your zip code ? Is less than 38614 ir greater than 71483

Q5 Are you a parent or caregiver for a child between the ages of 2-12?

○ Yes

○ No

Skip To: End of Survey if Are you a parent or caregiver for a child between the ages of 2-12? = No

© 2026 Fuster M et al. *JAMA Network Open*.

Q6 Have you ordered from or eaten at a restaurant in Baton Rouge or New Orleans (dependent upon survey location) with a child(ren) in the past 30 days?

- ○ Yes
- ○ No

Skip To: End of Survey If Have you ordered from or eaten at a restaurant in Baton Rouge with a child(ren) in the past 30 days? = No or If Have you ordered from or eaten at a restaurant in New Orleans with a child(ren) in the past 30 days? = No

© 2026 Fuster M et al. *JAMA Network Open*.

Q7 The following questions will ask about your experience eating out at restaurants. This can include eating at the restaurant, ordering delivery or take-out, or ordering in a drive-thru. This does NOT include meals purchased at supermarkets, food stands, or food trucks.

Q8 During the past 30 days, how often did your child(ren) eat meals from a restaurant? (Please include meals consumed at a restaurant or purchased at a restaurant as take-out or delivery).

- ◯ Once a month
- ◯ Twice a month (every other week)
- ◯ Once a week
- ◯ 2-3 times a week
- ◯ 4-6 times a week
- ◯ Once a day
- ◯ More than once a day

Q9 To ensure we have your full attention, please select "All of the above".

- ◯ Optimistic
- ◯ Positive
- ◯ Excellent
- ◯ Determined
- ◯ All of the above

Q10 Please think back to the last meal where your child(ren), aged 2-12, joined you at a restaurant in Baton Rouge or New Orleans (dependent upon survey location) for a meal that was ordered AND eaten there. We will ask about take-out and delivery in the next section.

Q11 Where did you eat? Please select the description that best matches the restaurant you visited.

- ◯ Fine dining (high-end establishments; dedicated courses)
- ◯ Casual dining (eg. Applebee's, Chili's; table service)
- ◯ Fast casual (eg. Panera Bread, Chipotle, Subway; counter service)
- ◯ Fast food (eg. McDonald's, Burger King, Domino's Pizza)
- ◯ Buffet-style (self-serve; “all you can eat” restaurant)
- ◯ Other (please specify): ____

Q12 What was the name of the restaurant where you last ate at in Baton Rouge or New Orleans (location of survey dependent) with your child(ren)? ________________________________

Q13 How many children between the ages of 2-12 were at this meal?

- ◯ One child
- ◯ Two or more children

Note: The next set of questions was asked once for those that dined with one child and twice for those that dined with two or more children. For those that dined with two or more children the questions were asked once about the oldest child dining at the meal and once about the youngest.

Q14 Please answer the following for the YOUNGEST/OLDEST/CHILD (dependent upon number of children) child between the ages of 2-12 present at the meal.

Q15 Child's Age: ________________________________

Q16 What is your child's sex?

- ◯ Female
- ◯ Male
- ◯ Prefer not to answer

Q17 Please select the option that best describes what the child ordered at this meal.

- ◯ Meal from the kid's menu
- ◯ Individual meal from the regular menu
- ◯ Child ate from my meal and/or another adult's meal
- ◯ Family meal ("family style")
- ◯ Other (please describe): ________________________________

Q18 What did the child drink? (Please select the MAIN drink consumed. If water was consumed, along with another drink, please select the beverage consumed aside from water)

- ◯ Only plain water (unsweetened, flat, still)
- ◯ Carbonated Water (unsweetened, eg. sparkling water, club soda, La Croix, Bubly, Topo Chico)
- ◯ Sweetened flavored waters (eg. Vitamin water)
- ◯ Sweetened carbonated soft drink (eg. Coca-Cola, 7-Up, Fanta)

© 2026 Fuster M et al. *JAMA Network Open*.

- ○ Diet drinks (eg. Diet Coke, Coke Zero, includes “lite” or “zero”)
- ○ Energy or sports drink (eg. Red Bull, Gatorade, Powerade)
- ○ Sweetened fruit drinks (eg. Sunny Delight, Lemonade, Capri Sun, Kool-Aid)
- ○ 100% Fruit Juice (eg. Honest Kids)
- ○ Sweetened tea (includes “lightly sweetened”)
- ○ Unsweetened tea
- ○ Sweetened coffee or coffee drinks (eg. Frappuccino’s, flavored coffees)
- ○ Non-fat or 1% plain milk
- ○ 2% or whole plain milk
- ○ Non-dairy milk substitute (eg. almond milk, soy milk, coconut milk, oat milk)
- ○ Sweetened or flavored milk (eg. chocolate milk)
- ○ Frozen drinks (e.g. slushies, frozen lemonade, iced-based)
- ○ Milkshakes (eg. milk-based frozen drinks)
- ○ No drink
- ○ Other (please describe): ____

Skip To: Q38 If What did the child drink? (Please select the MAIN drink consumed. If water was consumed, along wi... = No drink

Q19 Did the MAIN beverage have ice?

- ○ Yes
- ○ No

Q20 What size was the child’s MAIN drink? (Please use the following images to help you estimate as best as possible)

© 2026 Fuster M et al. *JAMA Network Open*.

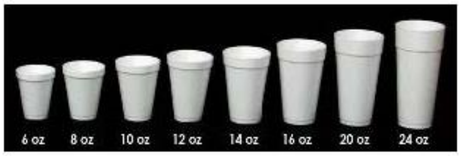

Note: Original survey included images of additional branded product examples of products found in restaurants, deleted for the purpose of publication.

- ◯ Extra Small (4-6 oz)
- ◯ Junior (8-12 oz)
- ◯ Small (13-16 oz)
- ◯ Medium (20-22 oz)
- ◯ Large (30-32 oz)
- ◯ Extra Large/ Jumbo (40+ oz)

Q21 How much of the MAIN drink did the child consume? If the child had water and another beverage, please answer this question for the other beverage (not water). (Please use the following images to help you estimate as best as possible)

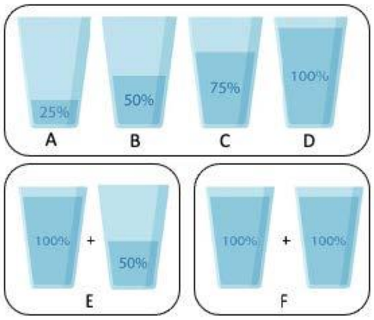

- ◯ A few sips/less than half (A)
- ◯ About half of the drink (B)
- ◯ Most of the drink (C)
- ◯ All of the drink (D)
- ◯ More than one drink but less than two (E)
- ◯ Two full drinks or more (F)

Q38 The following questions are about your last Baton Rouge or New Orleans (location of survey dependent) restaurant take-out or delivery meal purchased for a 2-12 year old child(ren).

Q39 How did you get the food?

- Delivery
- Take-out (order in restaurant and take home, or pick up from restaurant)
- Drive-thru

Q40 What was the name of the restaurant that you ordered from? ________________________

Q41 How many children between the ages of 2-12 were at this meal?

- One child
- Two or more children

Note: The next set of questions was asked once for those that dined with one child and twice for those that dined with two or more children. For those that dined with two or more children the questions were asked once about the oldest child dining at the meal and once about the youngest.

Q42 Please answer the following for the YOUNGEST/OLDEST/CHILD child between the ages of 2-12 present at the most recent take-out or delivery meal.

Q43 Child's Age: ____

Q44 What is your child's sex?

- Female
- Male
- Prefer not to say

Q45 Please select the option that best describes what the child ordered at this meal.

- Meal from the kid's menu
- Individual meal from the regular menu
- Child ate from my meal and/or another adult's meal
- Shared family meal ("family style")
- Other (please describe) ____

© 2026 Fuster M et al. *JAMA Network Open*.

Q46 What did the child drink? (Please select the MAIN drink consumed. If water was consumed, along with another drink, please select the beverage consumed aside from water)

- ◯ Only plain water (unsweetened, flat, still)
- ◯ Carbonated Water (unsweetened, eg. sparkling water, club soda, La Croix, Bubly, Topo Chico)
- ◯ Sweetened flavored waters (eg. Vitamin water)
- ◯ Sweetened carbonated soft drink (eg. Coca-Cola, 7-Up, Fanta)
- ◯ Diet drinks (eg. Diet Coke, Coke Zero, includes “lite” or “zero”)
- ◯ Energy or sports drink (eg. Red Bull, Gatorade, Powerade)
- ◯ Sweetened fruit drinks (eg. Sunny Delight, Lemonade, Capri Sun, Kool-Aid)
- ◯ 100% Fruit Juice (eg. Honest Kids)
- ◯ Sweetened tea (includes “lightly sweetened”)
- ◯ Unsweetened tea
- ◯ Sweetened coffee or coffee drinks (eg. Frappuccino’s, flavored coffees)
- ◯ Non-fat or 1% plain milk
- ◯ 2 % or whole plain milk
- ◯ Non-dairy milk substitute (eg. almond milk, soy milk, coconut milk, oat milk)
- ◯ Sweetened or flavored milk (eg. chocolate Milk)
- ◯ Frozen drinks (e.g. slushies, frozen lemonade, iced-based)
- ◯ Milkshakes (eg. milk-based frozen drinks)
- ◯ No drink

○ Other (please describe): ____

Q46 What did the child drink? (Please select the MAIN drink consumed. If water was consumed, along with another drink, please select the beverage consumed aside from water)

○ Only plain water (unsweetened, flat, still)

○ Carbonated Water (unsweetened, eg. sparkling water, club soda, La Croix, Bubly, Topo Chico)

○ Sweetened flavored waters (eg. Vitamin water)

○ Sweetened carbonated soft drink (eg. Coca-Cola, 7-Up, Fanta)

○ Diet drinks (eg. Diet Coke, Coke Zero, includes “lite” or “zero”)

○ Energy or sports drink (eg. Red Bull, Gatorade, Powerade)

○ Sweetened fruit drinks (eg. Sunny Delight, Lemonade, Capri Sun, Kool-Aid)

○ 100% Fruit Juice (eg. Honest Kids)

○ Sweetened tea (includes “lightly sweetened”)

○ Unsweetened tea

○ Sweetened coffee or coffee drinks (eg. Frappuccino’s, flavored coffees)

○ Non-fat or 1% plain milk

○ 2% or whole plain milk

○ Non-dairy milk substitute (eg. almond milk, soy milk, coconut milk, oat milk)

○ Sweetened or flavored milk (eg. chocolate Milk)

○ Frozen drinks (e.g. slushies, frozen lemonade, iced-based)

○ Milkshakes (eg. milk-based frozen drinks)

○ No drink

○ Other (please describe) : ____

Skip To: Q69 If What did the child drink? (Please select the MAIN drink consumed. If water was consumed, along wi... = No drink

© 2026 Fuster M et al. *JAMA Network Open*.

Q47 Did you purchase the drink from the same restaurant where you ordered the takeout or delivery?

◯ Yes

◯ No

Q48 Did the MAIN drink have ice?

◯ Yes

◯ No

Q49 What size was the child's MAIN drink? (Please use the following images to help you estimate as best as possible)

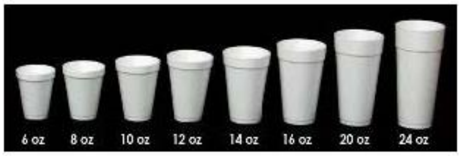

Note: Original survey included images of additional branded product examples of products found in restaurants, deleted for the purpose of publication.

◯ Extra Small (4-6 oz)

◯ Junior (8-12 oz)

◯ Small (13-16 oz)

◯ Medium (20-22 oz)

◯ Large (30-32 oz)

◯ Extra Large/ Jumbo (40+ oz)

Q50 How much of the MAIN drink did the child consume? If the child had water and another beverage, please answer this question for the other beverage (not water). (Please use the following images to help you estimate as best as possible)

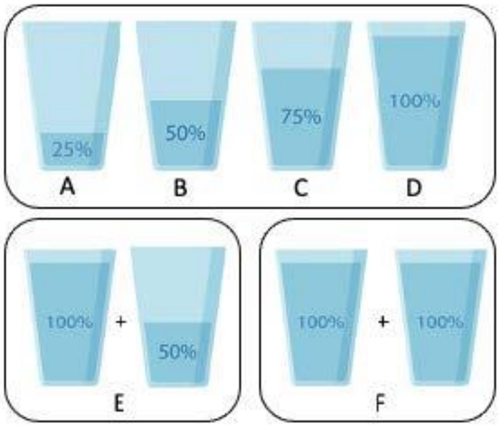

- ◯ A few sips/less than half (A)
- ◯ About half of the drink (B)
- ◯ Most of the drink (C)
- ◯ All of the drink (D)
- ◯ More than one drink but less than two (E)
- ◯ Two full drinks or more (F)

Q69 The following questions are to learn more about what you think about sugar-sweetened drinks. Please think of ANY drink with added sugar, such as: carbonated sodas/pop, fruit drinks (punch, concentrated lemonade), sweetened/flavored milk, fruit smoothies or shakes, energy and sports drinks, sweetened, flavored water, and sweetened teas.

Q70 During the last 30 days, in general, how often did your child(ren) consume sugar-sweetened drinks? Please include sodas/pop/carbonated sweetened drinks, fruit drinks, sweetened/flavored milk drinks, etc.

- ◯ Never/rarely
- ◯ Once a month
- ◯ 2-3 times a month
- ◯ 4-6 times a month
- ◯ 1 time per day

◯ 2 or more times per day

* Q71 In general, how often does your child(ren) consume sugar sweetened beverages at the following locations?

	Daily	Weekly	Monthly	Never
Home	◯	◯	◯	◯
School	◯	◯	◯	◯
Restaurants	◯	◯	◯	◯
Parks, sporting events or other recreational locations	◯	◯	◯	◯
Other (please specify):	◯	◯	◯	◯

* Q72 Which of the following are reasons why your child(ren) drink sugar sweetened beverages? Please check all that apply.

☐ My child(ren) like the carbonation/fizz/bubbles

☐ They make my child(ren) feel happy

☐ They are part of our routine at meals

☐ They are convenient to drink

☐ They are inexpensive/affordable

☐ Their friends drink them

☐ I do not trust our tap water to be safe to drink

☐ They like the taste

☐ Because I drink them

☐ Other reason(s) (please specify): ________________________________

© 2026 Fuster M et al. *JAMA Network Open*.

Q73 Please rate your level of agreement with the following statements.

Q74 Sugar-sweetened drinks are an important aspect of family meals

- Strongly Agree
- Agree
- Neutral
- Disagree
- Strongly Disagree

Q75 Restaurants should not offer sugar-sweetened drinks with children's meals

- Strongly Agree
- Agree
- Neutral
- Disagree
- Strongly Disagree

Q76 In 2023, the City of New Orleans implemented the Healthy Kids Meal Beverage Ordinance, requiring that New Orleans restaurants offering a children's meal with a drink only offer a choice of non-fat or 1% unflavored milk, 6.75 ounces of 100% fruit juice or plain, flat water as the beverages listed on the menu with any children's meal. This policy does not stop you from ordering beverages. Restaurants can offer other choices upon your request. Have you heard about this policy?

- Yes
- No

Display this question:

If In 2023, the City of New Orleans implemented the Healthy Kids Meal Beverage Ordinance, requiring... = Yes

* Q77 How did you hear about this policy?

- Social Media
- Media/news
- Friends or family

◯ Government official

◯ Work

◯ Restaurants

◯ Health department

◯ Medical professional

◯ Other (please specify): ____

* Q78 On a scale of 1 to 5 with 1 being not at all helpful and 5 being very helpful, how helpful do you think a policy like this could be in reducing sugar sweetened beverage consumption among kids?

	1 (Not at all helpful)	2 (Not very helpful)	3 (Neither helpful nor not helpful)	4 (Somewhat helpful)	5 (Very Helpful) (
Policy Helpfulness	◯	◯	◯	◯	◯

Q79 Why do you feel this way?

____

____

Q80 Do you support policies like this one?

◯ Yes

◯ No

Display this question:

If Do you support policies like this one? = Yes

Q81 Why do you support policies like this?

____

____

Display this question:

If Do you support policies like this one? = No

Q82 Why do you not support policies like this?

____

____

Q83 Thank you for your answers. The following questions are to learn more about you.

© 2026 Fuster M et al. *JAMA Network Open*.

* Q84 In your household within the past 12 months, have you worried about whether food would run out before you had enough money to buy more.

- Yes
- No

* Q85 In your household within the past 12 months, have there been times when the food you purchased ran out and you didn't have enough money to buy more.

- Yes
- No

Q86 What is your gender?

- Female
- Male
- Transgender
- Non-binary
- Other ____
- Prefer not to answer

Q87 What is your age? ____

* Q88 How many people, including yourself, live in your household? ________________________

* How many children under the age of 18 live in your household? _______________________

*Q90 Last year (2023) what was your total family income from all sources, before taxes?

- less that $30,000
- $30,000 - less than $40,000
- $40,000 - less than $50,000
- $50,000 - less than $60,000
- $60,000 - less than $70,000
- $70,000 to less than $80,000
- $80,000 to less than $90,000

© 2026 Fuster M et al. *JAMA Network Open*.

◯ $90,000 to less than $100,000

◯ $100,000 or more

◯ Don't know/Prefer not to answer

Display this question:

If Last year (2023) what was your total family income from all sources, before taxes? = less that $30,000

* Q91 And last year (2023) would you say your total family income before taxes was...

◯ Less than $5,000

◯ $5,000 to less than $10,000

◯ $10,000 to less than $15,000

◯ $15,000 to less than $20,000

◯ $20,000 to less than $25,000

◯ $25,000 to less than $30,000

Display this question:

If Last year (2023) what was your total family income from all sources, before taxes? = $100,000 or more

* Q92 And last year (2023) would you say your total family income before taxes was...

◯ $100,000 to less than $125,000

◯ $125,000 to less than $150,000

◯ $150,000 to less than $175,000

◯ $175,000 to less than $200,000

◯ $200,000 to less than $225,000

◯ $225,000 to less than $250,000

◯ $250,000 or more

Q93 What is your Race/Ethnicity? (please select all that apply)

▢ African American/Black

▢ Asian

▢ Latino/a/x or Hispanic

© 2026 Fuster M et al. *JAMA Network Open*.

- ☐ Native American
- ☐ Native Hawaiian or Pacific Islander
- ☐ White/Caucasian
- ☐ Prefer not to say
- ☐ Not specified (please describe): ____

Q94 What is your highest level of education?

- ○ Less than high school
- ○ High School / GED
- ○ Some College
- ○ Bachelor's Degree
- ○ Post-Graduate Education
- ○ Prefer not to say

© 2026 Fuster M et al. *JAMA Network Open*.
