# Supplementary material for: The New Orleans Healthy Default Beverage Policy and Beverage Ordering Among Children
Source: JAMA Netw Open. 2026 Jun 10;9(6):e2617749. doi: 10.1001/jamanetworkopen.2026.17749 (PMC13254729; doi:10.1001/jamanetworkopen.2026.17749)
Supplement: Supplement 2. — Data Sharing Statement [file jamanetwopen-e2617749-s002.pdf]

Data Sharing Statement

Fuster. The New Orleans Healthy Default Beverage Policy and Beverage Ordering Among Children. *JAMA Netw Open*. Published June 10, 2026.
doi:10.1001/jamanetworkopen.2026.17749

Data
Data available: Yes
Data types: Deidentified participant data
How to access data: Data will be provided under reasonable requests directly from the corresponding author, Dr. Melissa Fuster (mfuster@tulane.edu),
When available: With publication

Supporting Documents
Document types: None

Additional Information
Who can access the data: Researchers whose proposed use has been approved.
Types of analyses: For purposes related to the original study objective.
Mechanisms of data availability: Data will be made available after the investigative team approves the proposal with a signed data access agreement.
